# Carbohydrate restriction improves the features of Metabolic Syndrome. Metabolic Syndrome may be defined by the response to carbohydrate restriction

**DOI:** 10.1186/1743-7075-2-31

**Published:** 2005-11-16

**Authors:** Jeff S Volek, Richard D Feinman

**Affiliations:** 1 Human Performance Laboratory, Department of Kinesiology, University of Connecticut, Storrs, CT 06269-1110 USA; 2 Department of Biochemistry, SUNY Downstate Medical Center, Brooklyn, NY 11203 USA

## Abstract

Metabolic Syndrome (MetS) represents a constellation of markers that indicates a predisposition to diabetes, cardiovascular disease and other pathologic states. The definition and treatment are a matter of current debate and there is not general agreement on a precise definition or, to some extent, whether the designation provides more information than the individual components. We consider here five indicators that are central to most definitions and we provide evidence from the literature that these are precisely the symptoms that respond to reduction in dietary carbohydrate (CHO). Carbohydrate restriction is one of several strategies for reducing body mass but even in the absence of weight loss or in comparison with low fat alternatives, CHO restriction is effective at ameliorating high fasting glucose and insulin, high plasma triglycerides (TAG), low HDL and high blood pressure. In addition, low fat, high CHO diets have long been known to raise TAG, lower HDL and, in the absence of weight loss, may worsen glycemic control. Thus, whereas there are numerous strategies for weight loss, a patient with high BMI *and *high TAG is likely to benefit most from a regimen that reduces CHO intake. Reviewing the literature, benefits of CHO restriction are seen in normal or overweight individuals, in normal patients who meet the criteria for MetS or in patients with frank diabetes. Moreover, in low fat studies that ameliorate LDL and total cholesterol, controls may do better on the symptoms of MetS. On this basis, we feel that MetS is a meaningful, useful phenomenon and may, in fact, be operationally defined as the set of markers that responds to CHO restriction. Insofar as this is an accurate characterization it is likely the result of the effect of dietary CHO on insulin metabolism. Glucose is the major insulin secretagogue and insulin resistance has been tied to the hyperinsulinemic state or the effect of such a state on lipid metabolism. The conclusion is probably not surprising but has not been explicitly stated before. The known effects of CHO-induced hypertriglyceridemia, the HDL-lowering effect of low fat, high CHO interventions and the obvious improvement in glucose and insulin from CHO restriction should have made this evident. In addition, recent studies suggest that a subset of MetS, the ratio of TAG/HDL, is a good marker for insulin resistance and risk of CVD, and this indicator is reliably reduced by CHO restriction and exacerbated by high CHO intake. Inability to make this connection in the past has probably been due to the fact that individual responses have been studied in isolation as well as to the emphasis of traditional therapeutic approaches on low fat rather than low CHO.

We emphasize that MetS is not a disease but a collection of markers. Individual physicians must decide whether high LDL, or other risk factors are more important than the features of MetS in any individual case but if MetS is to be considered it should be recognized that reducing CHO will bring improvement. Response of symptoms to CHO restriction might thus provide a new experimental criterion for MetS in the face of on-going controversy about a useful definition. As a guide to future research, the idea that control of insulin metabolism by CHO intake is, to a first approximation, the underlying mechanism in MetS is a testable hypothesis.

## Introduction

An association between obesity, diabetes, cardiovascular disease and hypertension has been recognized for some time. Reaven's 1988 Banting lecture is generally considered a turning point in codifying a unifying principle under the name of MetS or Syndrome X (Reviews: [1-7]. Although there is no universally accepted definition or mechanism, (Table 1) a rough common denominator is the set of five features: obesity (high body weight, BMI and/or waist circumference), high glucose and insulin levels, low HDL, high TAG and high blood pressure. Involvement of insulin resistance is generally a common feature and a likely causative agent for at least some of the symptoms. A subset of these metabolic markers, the TAG:HDL ratio, has been proposed as a simple marker for identifying insulin resistance [8]. It has recently been questioned whether the risk attributed to MetS is greater than the sum of the individual symptoms [9] and, ironically, Reaven has taken the "Con" side on a debate on the viability and diagnostic usefulness of the concept [10,11]. Nonetheless, there seems little controversy on the inherent potential for risk in the individual components.

**Table 1:** NCEP-ATP III and WHO Definitions of Metabolic Syndrome. Other definitions in references [1], [4] and [90].

ATP III Definition
Any three or more of the following criteria:
a) Waist circumference: >102 cm in men, >88 cm in women
b) Serum triglycerides: ≥150 mg/dL
c) HDL-cholesterol: <40 mg/dL in men, < 50 mg/dL in women
d) Blood pressure: ≥130/85 mm Hg
e) Serum glucose: >110 mg/dL
**WHO Definition**
Diabetes or IFG or IGT or insulin resistance, plus at least two of the following criteria
a) Waist-to-hip ratio: >0.90 in men, >0.85 in women
b) Serum triglycerides: >150 mg/dL *or *HDL-cholesterol: <35 mg/dL in men and <40 mg/dL in women
c) Blood pressure: >140/90 mmHg
d) Urinary albumin excretion rate > 20 μg/min *or *albumin/creatinine ratio >30 mg/g

In reading recent reviews of low CHO diets [12,13], we were struck by the fact that the symptoms of MetS are precisely the ones targeted by diets that restrict CHO. This effect is not entirely surprising since it has been known that dietary CHO tends to raise glucose, insulin, and TAG and lower HDL and conversely, replacing CHO with monounsaturated fat or with fat and protein improves glycemic control and dyslipidemia expressed as elevated TAG and lowered HDL. Nevertheless, most formal guidelines and clinical papers have not emphasized CHO restriction as a viable approach to treating MetS or the individual components [14,15] and although several authors have indicated an association between MetS and CHO restriction in passing [5,13,16,17] the explicit connection has not been made.

In this study we have isolated five features that are common to almost all definitions. Waist circumference is probably the currently preferred measure for obesity, but most of the literature provides data on body mass and we have used that measure. We have collected information in the literature supporting the notion that these symptoms are specifically ameliorated by reduction in dietary CHO, and to the extent that they have been directly compared, low CHO strategies appear to have an advantage over low fat diets or simple calorie reduction. We conclude that response to CHO restriction may be an operational definition for MetS and that a likely mechanism is the control of insulin metabolism. Finally, we side with those who maintain that MetS is a real thing in the sense that the concomitant appearance of several symptoms may provide different recommended strategies than the isolated factors. It is important to point out, however, that MetS is not a disease but a complex of markers and practitioners may decide that LDL or other factors are more important for individual patients and in these cases other therapies will be appropriate. On the other hand, reliance on LDL as a prime indicator must be tempered by the importance of LDL particle phenotypes which, in turn, correlate with TAG and HDL levels, a subset of MetS markers.

### CHO restriction for weight loss

It is sometimes stated that MetS is caused by obesity [1]. In our view, this is only one of several possible theories and would assume that we know the causes of obesity. It is at least plausible that obesity and the features of MetS arise in parallel from disruptions of insulin metabolism (possibly a consequence of high insulin due to chronic high dietary CHO). Also a high prevalence of so called metabolically obese-normal-weight individuals with MetS has long been known [18]. In any case, it is generally agreed that the first line of attack against MetS or frank diabetes should be reduction in body mass. The method for attaining this weight loss, however, is more controversial. Studies in the literature imply that a low fat diet is a kind of standard although much recent evidence has indicated the value of strategies based on carbohydrate restriction. Whereas low fat diets for calorie reduction can undoubtedly be effective for many people, we feel that they cannot be taken as an established standard; to our knowledge, there has never been a long term study where a low fat diet was instituted in the absence of confounding features such as cessation of smoking and exercise. Also, fat restriction *per se *does not enhance long-term (one year or longer) weight loss or prevent regain of weight [19] and the record of compliance is modest at best [20]. Most important, the fact that, in the obesity and diabetes epidemic, fat consumption went down (for men, the absolute amount) and carbohydrate consumption went up [21,22], means that other approaches should be considered. Insofar as isocaloric comparisons have been made, low CHO diets do at least as well, and usually better, than low fat diets (see below). Most striking, in *ad lib*. trials, subjects on low CHO diets show a spontaneous reduction in calories without any dissatisfaction [23-25], a goal that is universally considered desirable but generally recognized as difficult to impose by cognitive admonitions on calorie restriction *per se *[26]. In general, published data support the idea that low CHO diets are at least as effective as other weight reduction methods. Further, experimental results show an improvement in lipid outcomes (discussed below), no damage to normal kidneys [27], the potentially beneficial rather than deleterious effects of ketone bodies [28-30] and the prevalence of strategies based on low glycemic index [31,32] or reduction of refined CHO or sweets, all approximations of low CHO diets.

Despite increased acceptance of low carbohydrate regimens, it is important to point out that there is a tendency to equate any kind of carbohydrate restriction with the popular Atkins diet [33] and to equate the Atkins diet with a recommendation for high fat and with high saturated fat, in particular. There are, however, many strategies for reducing carbohydrate intake both clinically and in popular diets [34,35]. Whereas high fat is permitted on the Atkins diet and other low carbohydrate diets it is not specifically recommended; as noted above, at least three published studies [23-25] and much anecdotal evidence suggests that, in practice, the major effect is reduction in carbohydrate intake with limited replacement with either fat or protein. In addition, although a deleterious effect of saturated fat, at least in the absence of CHO control, is established [36,37], it has been known since the Seven Countries study [38] that total dietary fat does not correlate with cardiovascular risk and the two effects should not be confused [36,37]. In any case, no particular diet is recommended here; the studies cited include all kinds of interventions, and the underlying rationale is the effect of carbohydrate on insulin. The principle espoused here should be evaluated on this basis.

It seems that a prudent statement of the state of affairs would be that, at this point, dieters have many strategies, none perfect, for weight loss and CHO restriction of some kind is one of them.

### CHO restriction and MetS

Our argument in the following exposition is that a patient presenting with a high BMI or large waist line has several options for weight loss. Many factors, including physician experience, ethnic background, personal taste and genetic profile, will determine the first one to be tried. A patient presenting with high BMI *and *high TAG may have a clear best strategy because of the known benefit of CHO reduction and the accepted deleterious effect of high CHO intake. Data from the literature suggests that a patient with more than two of the symptoms of MetS or the particular combination of high TAG/HDL ratio should clearly try CHO restriction as a first strategy. Conversely, the patient with high BMI and high LDL might sensibly try a low fat strategy first. We provide a summary of cases in which CHO restriction is beneficial in the treatment of MetS or its individual symptoms. The review is meant to be representative rather than comprehensive but we think that the wide variety of cases studied and the range of conditions against a background of accepted effects of carbohydrate on the relevant parameters, provides a strong case for our thesis. In addition, the generally consistent benefit of CHO restriction allows a possible further basis for identifying the common thread in MetS if such truly exists.

### CHO restriction improves symptoms of MetS

Table 2 (Table 2) shows the results of single arm studies in which CHO-restricted diets of various compositions and duration were used (for summary of details see, e.g., [39-41]). The regimens include very low CHO ketogenic diets (< 50 g/d) and encompasses subjects that were overweight, presented with symptoms of MetS, or were diabetic. It is clear that CHO restriction is effective in relieving these symptoms. Noteworthy is the recent study of Boden [23] which, while short in duration, carefully measured relevant parameters in patients with diabetes. Patients in this study spontaneously decreased food intake to a substantial degree, were satisfied with the diet, did not show substantial water loss and several were able to reduce or terminate medication.

**Table 2:** 

				CHANGE						
				
Reference	#	Subjects	Duration	CHO (g/d)	weight (%)	HDL (%)	TAG (%)	TAG/HDL (%)	Glucose (%)	Insulin (%)
Rickman *et al*. 1974	1	Normal Weight Men/Women	3–17 d	7	***-4.9***		***-11.4***			
LaRosa *et al*. 1980	2	Obese Men/Women	8 wk	6	***-8.3***	-5.7	***-32.6***	***-29.9***		
Phinney *et al*. 1980	3	Obese Men/Women	6 wk	<20	***-11.8***		***-24***		***-16.3***	***-57.3***
Phinney *et al*. 1983	4	Normal Weight Men	4 wk	<20	0.2	0	***-26***		***-7.7***	***-23.3***
Newbold, 1988	5	Men	3–12 mo			***9.6***	***-34.8***	***-40.5***		
Volek *et al*. 2000	6	Normal Weight Men	8 wk	39	***-5.4***	***10.0***	***-54.9***	***-56.9***	***-3.4***	***-28.0***
Sharman *et al*. 2002	7	Normal Weight Men	6 wk	46	***-2.8***	***11.5***	***-33***	***-39.9***	***-0.2***	***-34.2***
Meckling *et al*. 2002	8	Obese Women	8 wk	71	***-6.1***	***4.3***	***-40.3***		***-4.1***	***0.0***
Westman *et al*. 2002	9	Obese Men/Women	6 mo	Ad Lib	***-10.3***	***19.2***	***-43.1***	***-53.6***		
Dashti *et al*. 2003	10	Obese Men/Women	12 wk	20 to 30	***-13.4***	***8.3***	***-50***	***-53.9***	***-37.1***	
Hays *et al*. 2003	11	Obese Men/Women w/ CVD	6 wk	Ad Lib	***-5.2***	-2.9	***-39.9***	***-38.1***	***-7.4***	***-30.5***
		Obese Women PCOS	24 wk	Ad Lib	***-14.3***	***0.4***	***-18.5***	***-18.8***	5.7	***-49.6***
		Obese Women Reactive Hypoglycemia	52 wk	Ad Lib	***-19.9***	***3.4***	***-13.3***	***-33.2***		
Dashti *et al*. 2004	12	Obese Men/Women	24 wk	40	***-14.2***	***20.4***	***-60.4***	***-67.1***	***-22.6***	
Boden *et al*. 2005	13	Obese/diabetic men/women	14 days	21	***-1.8***	***-2***	***-35***	***-33.8***	***-16.0***	

### Improvement is seen in the absence of weight loss

Since it is known that weight loss generally improves MetS, it is important to ask whether beneficial metabolic responses to low CHO diets are dependent on weight loss. The question was specifically addressed by Volek's group [42,43] in normal-weight men and women encouraged to maintain their weight and by Allick and colleagues in patients with diabetes mellitus type 2 [44] (Table 3). The studies in normal weight women [43] and type 2 diabetics [44], in particular, used a cross-over experimental design removing the confounding effect of group differences. These studies were also well-controlled. In the case of Allick, formula was used and, in the studies by Volek, compliance was documented by measuring elevation of serum and urine ketones, thereby eliminating dietary reporting errors as a confounding factor. Improvement in the TAG/HDL ratio ranged from 40 to 55%. In summary, a low CHO regimen clearly improves MetS relative to low fat diets even in the absence of weight loss.

**Table 3:** 

					CHANGE						
					
Reference	#	Subjects	Duration	Diet	CHO (g/d)	weight (%)	HDL (%)	TAG (%)	TAG/HDL (%)	Glucose (%)	Insulin (%)
Sharman *et al.*2002	1	Normal Weight Men	6 wk	Ketogenic	46	***-2.8***	***11.5***	***-33.0***	***-39.9***	***-0.2***	***-34.2***
			6 wk	Low-Fat	271	***0.5***	***0.0***	***-5.3***	***-5.3***	***1.8***	***13.0***
Volek *et al*. 2003	2	Normal Weight Women	4 wk	Ketogenic	43	***-2.0***	***32.0***	***-30.2***	***-47.2***	-1.9	***11.6***
			4 wk	Low-Fat	249	***-1.3***	***-7.7***	***3.8***	***0.5***	-5.3	***18.7***
Allick *et al*. 2004	3	Type 2 Diabetics	2 wk	Ketogenic	0	0	***23.5***	***-43.9***	***-55***	***-16.9***	***-16.7***
			2 wk	Low-Fat	775						

**Figure 1:**
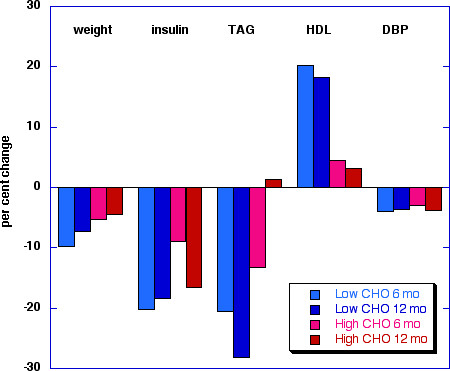
**Comparison of features of Metabolic Syndrome on low carbohydrate vs. high carbohydrate diets**. Data from reference [45].

In addition to studies in which weight maintenance was a feature of experimental design it is important to consider data reported by Foster [45] (Figure 1, (Table 4), who compared low CHO and LF diets. It is widely quoted that the low CHO diet is better at 6 months but that there is no difference in the diets at 12 months. However, it has been pointed out [12] that the particular form of low CHO diet used (Atkins diet) allowed increases in CHO consumption as the trial progressed indicating that it is likely this reintroduction of CHO that predisposes to long-term regain in weight. Most notable in this study, is that the improvement in lipid profile persisted (Figure 1, (Table 4) even after the effect on weight loss disappeared.

**Table 4:** 

					CHANGE							
					
Reference	#	Subjects	Duration	Diet	CHO (g/d)	weight (%)	HDL (%)	TAG (%)	TAG/HDL (%)	Glucose (%)	Insulin (%)	DBP (mm Hg)
Brehm *et al*. 2003	1	Obese Women	6 mo	Low-CHO	41–97	***-9.3***	***13.4***	***-23.4***	***-32.4***	***-9.1***	-14.8	***-5***
				LF	163–169	***-4.2***	***8.4***	***1.6***	***-6.3***	***-4.0***	-23.0	***-1***
Sondike *et al*. 2003	2	Overweight Adolescents	12 wk	Low-CHO	37	***-10.7***	***8.7***	***-40.5***	***-45.2***			
				LF	154	***-4.1***	***4.2***	***-5.4***	***-9.2***			
Samaha *et al*. 2003	3	Obese Men/Women	6 mo	Low-CHO	150	***-4.5***	***0.0***	***-20.2***	***-20.2***	***-8.6***	**-27.3**	
				LF	201	***-1.4***	***-2.4***	***-4***	***-1.6***	***-1.6***	**5.6**	
Foster *et al*. 2003	4	Obese Men/Women	1 yr	Low-CHO	ad lib	***-7.3***	***18.2***	***-28.1***	***-29.5***			
				LF	ad lib	***-4.5***	***1.4***	***0.7***	***-2.6***			
Volek *et al*. 2004	5	Overweight Women	4 wk	Low-CHO	29	***-3.9***	***1.3***	***-23***	***-28.3***	***-3.8***	***-8.8***	
				LF	186	***-1.4***	***-8.6***	***-11.2***	***-4.2***	***1.3***	***23.2***	
Sharman *et al*. 2004	6	Overweight Men	6 wk	Low-CHO	36	***-5.6***	***-3.3***	***-44.1***	***-42.3***	***-5.8***	***-41.5***	
				LF	224	***-3.6***	***-6.6***	***-15***	***-8.3***	***-5.2***	***-28.1***	
Brehm *et al*. 2004	7	Obese Women	4 mo	Low-CHO	69	***-10.8***	***16.3***	***-37.3***	***-46.1***			***-9***
				LF	174	***-6.8***	***4.5***	***-10.3***	***-14.2***			***-3***
Meckling *et al*. 2004	8	Obese Men/Women	10 wk	Low-CHO	59	***-7.7***	***12.2***	***-29.4***	***-37.1***	-8.0	***-28.7***	***-6.1***
				LF	225	***-7.4***	***-15.4***	***-25.4***	***-11.8***	-10.2	***-3.3***	***-5***
Stern *et al*. 2004	9	Obese Men/Women	1 yr	Low-CHO	120	***-3.9***	***-2.8***	***-28.6***	***-26.8***			
				LF	230	***-2.3***	***-12.3***	***2.7***	***29.6***			
Yancy *et al*. 2004	10	Obese Men/Women	24 wk	Low-CHO	30	***-12.3***	***9.8***	***-47.2***	***-51.8***			***-6***
				LF	198	***-6.7***	***-2.9***	***-14.4***	***-12.1***			***-5.2***
Aude *et al*. 2004	11	Obese Men/Women	12 wk	Low-CHO	ad lib	***-6.2***	***-2.6***	***-23.2***	***-21.1***			
				LF	ad lib	***-3.4***	***-7***	***-10.5***	***-3.8***			
Seshadri *et al*. 2004	12	Obese Men/Women	6 mo	Low-CHO	113	***(-8.5)***	-2.4	***-7.4***			***-40***	
				LF	198	***(-3.5 kg)***	-2.4	***-2.3***			***11.2***	
McAuley *et al*. 2004	13	Obese Women	8 wk	Low-CHO	41	***-6.9***	***0.9***	***-38.8***	***-44.2***	***-5.9***	***-39.3***	
				LF	172	***-4.4***	***-6***	***-17.5***	***-15.1***	***-0.1***	***-28.4***	
				Mod-PRO	130	***-5.8***	***-4.1***	***-33.9***	***-31.8***	***-3.9***	***-24.4***	
Dansinger *et al*. 2004	14	Obese Men/Women	2 mo	Low-CHO	103	-4.7	***8.8***	***-27.6***	***-26.20***	***-10***	***-29.5***	
				Mod-PRO	158	-4.6	***4.6***	***-34***	***-30.50***	***-9.3***	***-27.7***	
				LF	183	-4.3	***-0.6***	***-7.1***	***-5.60***	***-5.7***	***-11***	
				UltraLF	230	-4.9	***-10.9***	***-0.6***	***8.40***	***-3.5***	***-7.7***	

### In *ad lib*. comparisons low CHO diets do better than low fat diets for weight loss and MetS

At this point, we have established that CHO restriction improves MetS and that this can be independent of weight loss. Weight loss can, of course, occur with low fat diets and we next consider the extent to which one or the other strategy is more effective. (Table 4) summarizes results of several studies in the literature demonstrating that low CHO diets generally do better than low fat diets in *ad lib*. comparisons. Although there is great variability, a pattern of better responses on very low CHO is evident. It is notable that Samaha et al studied a population in which 39 % had diabetes and 43% had MetS [46].

Figure 1 shows data from the study of Foster, et al. [45] and, as noted above, despite the relative similarity in weight loss, the markers of MetS were more favorable in the low CHO arm than the LF arm.

### In isocaloric comparisons, low CHO diets do better than LF diets for weight loss

Because weight reduction is considered the first line of attack in MetS or frank diabetes it is worth considering the record of low CHO diets on this parameter alone. It is generally agreed that the major effect of a low CHO diet is a spontaneous reduction in calories. In studies mentioned above, subjects did not significantly increase fat or protein intake but merely removed CHO from their diets [23-25]. Foster and Samaha also attributed the better performance of low CHO arm to decreased caloric intake, although this was not actually measured.

Beyond spontaneous caloric reduction, however, it has been shown that the macronutrient composition of the diet can affect the efficiency of energy utilization and greater efficacy, the so-called metabolic advantage, of low CHO diets compared to LF diets has been the subject of several reports (Reviews: [39,41]. It has long been argued that there must be some mistake because it is physically impossible and would violate the laws of thermodynamics. We have shown this argument is based on misunderstanding of the laws of thermodynamics [39,47-49] and the effect of variable efficiency is now better accepted [50,51]. The precise conditions that allow the so-called metabolic advantage to occur are not known although Cornier, *et al*. [51] have suggested that those subjects with insulin resistance will show a metabolic advantage on a low CHO diet whereas those who are insulin sensitive do better on low fat. This is consistent with the proposal here, namely that MetS, where insulin-resistance is generally considered a major component, can be defined by the response to CHO restriction. The study of Cornier, *et al*. [51] had only a small number of subjects and the low CHO arm was not particularly low (40%) but their theory follows from the general rationale of the effect of CHO on energy efficiency. The factors that determine whether a metabolic advantage can play a role in a CHO restricted diet is unknown but given that the insulin resistance association is reasonable, it would seem that some form of CHO restriction is one of the standard, if not preferred attacks on obesity where MetS is suspected.

**Figure 2:**
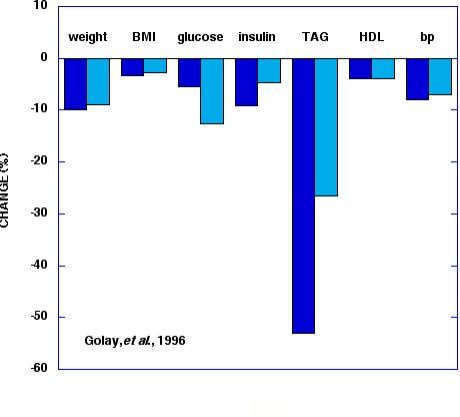
**Per cent change in response to diet**. Low carbohydrate (dark blue 25 % CHO) and low fat diets(light blue: 45 % CHO). Data from Golay, et al. [52].

Figure 2 shows data from Golay, *et al*. [52] This study is widely quoted as an example of how weight loss is independent of macronutrient composition; although the low CHO arm did better in weight loss, this was judged not significant. This may well be an experiment in which metabolic advantage does not occur – the effect is only possible, not required [39]. It is clear, however, from the figure that there is improvement in TAG and insulin and Golay's conclusion was that "...considering the greater improvement of fasting blood insulin, the glucose/insulin ratio and blood triglyceride, the low carbohydrate diet (25%) could be more favourable in the long-term [52]."

**Figure 3:**
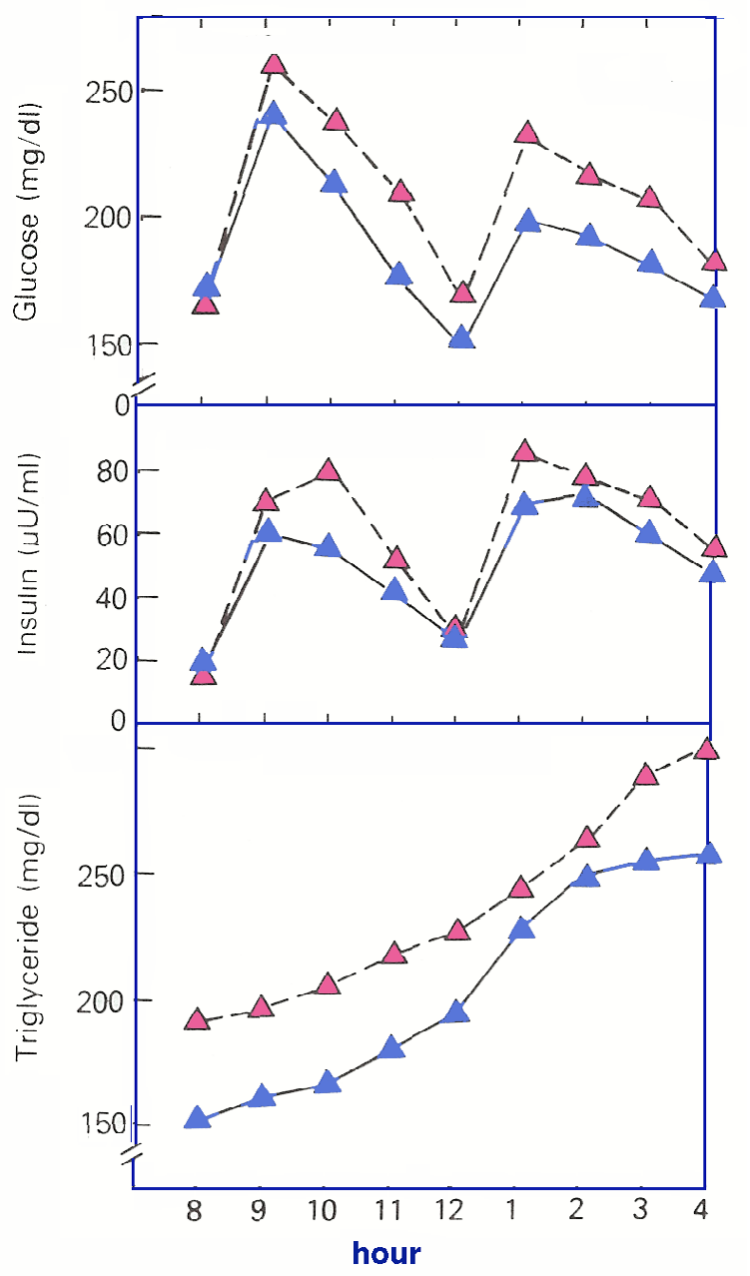
**Effect of carbohydrate on parameters of Metabolic Syndrome**. Comparison of 40% CHO (blue) and 55% (red) CHO diets. Data from [54].

### Is this new?

In Edgar Allan Poe's detective story The Purloined Letter, the police search the apartment for a missing blackmail note [53]. In the end, Poe's detective, Auguste Dupin reveals that it had been in plain view on the fireplace all along. The effect of CHO reduction on the symptoms of MetS has, in fact, been visible for some time. In a classic review in 1986, Reaven demonstrated the relative effects of 40% and 55% CHO [54]. Figure 3 shows data from that study: day long glucose, insulin and TAG levels were improved by the low CHO diet. Of interest, is that fasting glucose is not different on the two diets but there is a clear difference in the time course, common to several low CHO interventions (Table 3)). Reaven's experiment suggests that a nutritional approach to MetS is possible by lowering dietary CHO. The experiment should sensibly have spurred research to see whether still lower CHO had further beneficial effect. It took many years, however, before this was done.

**Figure 4:**
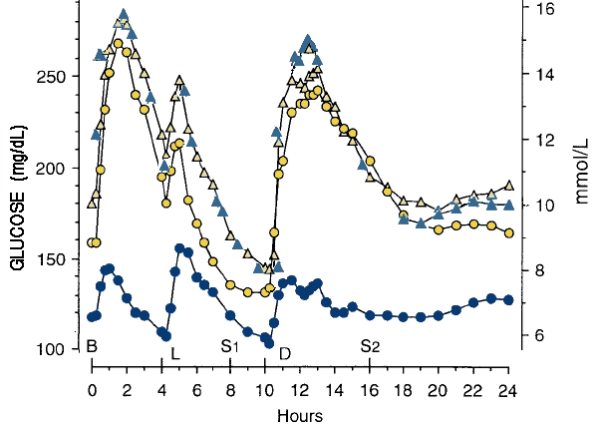
**Effect of diet on plasma glucose**. Mean plasma glucose concentration before (triangles) and after 5 weeks on control diet (yellow circles: (CHO:fat:protein = 55:30:15)) or 5 weeks on lower carbohydrate diet (blue circles: (20:50:30)). Meal points are Breakfast (B), lunch (L) and dinner(D) plus 2 snacks (S1, S2). Data from reference [57].

### Substitution of protein for CHO improves MetS

Despite the evidence from Reaven's experiment, a barrier to progress in understanding the role of CHO restriction was the accepted idea that high fat was unhealthy. At the same time, it was thought that an increase in protein would be deleterious for type 2 diabetics because of the increase in glucose due to gluconeogenesis. Nuttall and Gannon have summarized the history of this problem and work in their lab showed that, in fact, glycemic control was enhanced by a diet that was 40 % CHO, with protein replacing part of the carbohydrate [55,56]. Most recently, this group has shown the benefit of a 20 % CHO diet with higher protein [57]. Results in Figure 4 show a pattern similar to Reaven's but much more dramatic. Similar striking differences in the control of insulin and TAG were also demonstrated.

**Figure 5:**
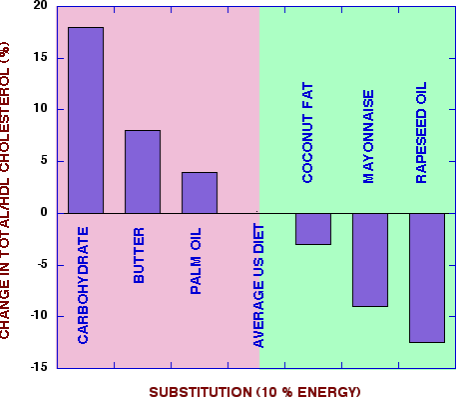
**Changes in cholesterol/HDL for substitution for fat**. Fat in the average American diet was substituted with the indicated substances at 10 % of energy. Data from reference [62].

### Explicit low-fat/high CHO interventions exacerbate MetS

In the approach taken here, we see dietary fat as playing a largely passive role (not withstanding differences between different fats) and the disposition of dietary fat is controlled by insulin and other hormones that, in turn, depend on dietary CHO which we take as the controlling variable. Thus, characterizing a low CHO diet as high fat [58,59] ignores the question about the underlying mechanism; we have recently raised the question of whether "high fat" is a meaningful description in the absence of information about CHO [60]. In the cases we discuss next, the focus is interventions described or designed as low fat. Our point here, however, is that although low fat diets exacerbate the markers of MetS, it is likely the high CHO rather than the fat level that is important.

A very influential paper by Garg, et al. [61] describes a four center randomized study of patients with type 2 diabetes receiving glipizide treatment. The study compared diets where monounsaturated fat was substituted for CHO or vice-versa. Because of its importance, we quote from this article. The rationale for the study, according to the authors is that:

"Compared with diets rich in saturated fats, low-fat, high-carbohydrate diets are reported to reduce serum low-density lipoprotein (LDL) cholesterol levels. Recent studies, however, suggest the high-carbohydrate diets may accentuate hypertriglyceridemia, reduce serum high-density lipoprotein (HDL) cholesterol concentration, and may even worsen hyperglycemia and/or raise plasma insulin levels."

In short, the question is whether high CHO diets worsen MetS. The conclusions of the paper state:

"The study confirms that HC (high carbohydrate diets) increase plasma TAG levels and increase VLDL-C concentrations in NIDDM patients. In this study the HC diet raised fasting plasma triglyceride levels and VLDL-C concentrations by 24 % and 23 % respectively compared with the HMUF diet. Furthermore, daylong levels of plasma triglycerides were also elevated on the high-carbohydrate diet. Consistent with the results of previous studies, plasma levels of total cholesterol and LDL cholesterol were not different on the two diets in this study. The study, therefore substantiates the fact that high carbohydrate diets offer no advantage in lowering LDL levels in NIDDM patients compared with high-fat diets that are low in saturated fats."

### The general case: substitution of fat for CHO improves MetS

The substitution of fat for CHO is, in fact, generally beneficial for MetS. In a recent meta-analysis, Mensink, *et al*. [62] showed the effect of substitution of different fat sources or carbohydrate for the fat in the average US diet at 10% of energy. The conclusion was that substitution of carbohydrate had the most unfavorable response on the total cholesterol to HDL ratio, significantly worse than butter or palm oil (Figure 5).

### Summary of review and hypothesis to this point

We have summarized work in the literature showing that low CHO interventions improve the markers of MetS in normal subjects, patients with MetS and diabetics. In comparative studies, they are at least as effective as low fat diets for weight loss and, tend to show better improvement in the other markers of MetS. Isocaloric studies similarly support the idea that the markers of MetS respond preferentially to low CHO diets.

The state of accepted scientific thinking for the world at large is unknown but we have made the case that it is an acknowledged principle that low CHO diets tend to reduce TAG, raise HDL and improve glycemic control whereas LF/high CHO diets tend to have the opposite effect. Perhaps the strongest indication that such an idea is generally accepted is the paper by Rock, et al [63] where the effect of low fat diets in cancer patients was studied. To demonstrate compliance with the low fat recommendations, the authors showed increased TAG and reduced HDL levels. These effects were judged not significant enough to cause a risk for CVD but demonstrate that low fat (higher CHO) diets point in that direction.

We reiterate that this article is not meant to make recommendations – for which many factors must be considered – but rather to show the association between CHO restriction and improvement in symptoms of MetS. Many low fat interventions have successfully reduced LDL, an established risk factor for CVD. If our hypothesis is correct, however, these same interventions should worsen the features of MetS. To some extent this is established from the principles noted above, but we provide an example from a well done experiment in the literature to support this corollary. Finally, in considering the dialectic of treating MetS with CHO restriction vs. high LDL, with low fat diets, it is important to consider both individual variation and the role of LDL particle size. We consider that last.

### Studies of low-fat diets

The most salient feature of the obesity epidemic from the standpoint of food consumption is the dramatic increase in CHO intake and the reduction in fat intake (for men, the absolute amount). To our knowledge, the decreased fat intake has not been accompanied by reduction in the incidence of CVD in unmedicated population. These data suggest that low fat diet recommendations *per se *are not likely to help MetS. If the fundamental idea proposed here is correct, then experimental interventions targeting lower fat that concomitantly raise CHO should, in fact, have a deleterious effect on the markers of MetS. Again, one will have to decide if the symptoms of MetS are more important than LDL or total cholesterol which are typically reduced on LF diets in the unmedicated population.

### Delta-1 Study

The Delta-1 study is one of the very well done trials involving a large number of participants [64]. The goal was to determine "the effects of reducing total fat and saturated fat" although this is slightly misleading in that only saturated fat was reduced and any reduction in total fat was a consequence of this. The randomized and balanced diets that were compared all contained approximately 15 % of calories as protein. Other macronutrients were as shown in Table 5. (Table 6) shows the outcomes for all groups on lipids in the Delta-1 study. The results indicate that LDL is significantly reduced although only SEM is given so that it is not possible to know the range of responses of subjects. As a group, however, there were step-wise reductions in LDL and HDL going from the average American diet (AAD) to Step 1 to the low saturated fat diet. There is, as well, a corresponding increase in TAG and hence the TAG/HDL ratio. The authors concluded that the reduction in LDL should be associated with 10% to 20% reductions in cardiovascular disease in the population. Other authors have argued, however, that the effect of the 13% higher HDL seen on the AAD might be associated with a 36% reduction in the risk of death from coronary disease or of myocardial infarction [65]. Again, the purpose here is not to decide on the relative risk attached to different markers but only to point out that the markers for MetS provide another side to the story. An important follow-up in the Delta study to determine HDL subpopulations [66], showed that the more *anti*-atherogenic HDL_2 _particles were, in fact, decreased by reductions in saturated fat.

**Table 5:** Macronutrient composition of diets in the Delta-1 study. Data from reference [64].

Diet	CHO (%)	total fat (%)	SFA (%)	MUFA (%)	PUFA (%)
**Average American diet (AAD)**	48	34	15	13	7
**Step 1**	55	29	9	13	7
**Low Saturated Fat**	59	25	6	12	7

**Table 6:** 

	Ave American Diet vs. Step 1				Ave American Diet vs. Low Sat Fat			
Ginsberg, *et al*.,1998 reference [64]	AAD	Step 1	delta	% change	pre	post	delta	% change
Total cholesterol	202.1	191	-11.1	-5.5	202.1	183.4	-18.7	-9.3
LDL	131.4	122.2	-9.2	-7.0	131.4	116.9	-14.5	-11.0
TAG (mmol/l) antiln (log)	85.1	92.4	7.3	***8.6***	85.1	93	7.9	***9.3***
HDL (mmol/l)	52.2	48.5	-3.7	***-7.1***	52.2	46.2	-6.0	***-11.5***
Total/HDL	4.1	4.16	0.1	***2.2***	4.1	4.21	0.1	***3.4***
								
TAG/HDL (arbitrary units)	1.6	1.9	0.3	***16.9***	1.6	2.0	0.4	***23.5***

The overall conclusion is that "dietary changes suggested to be prudent for a large segment of the population will primarily affect the concentrations of the most prominent antiatherogenic HDL subpopulation. However, the simultaneous reduction in the atherogenic LDL subpopulation will most likely offset any potential negative effect on cardiovascular risk." As noted above, the decision as to "most likely" outcome must rest with individual patients and physicians.

### Role of individual responses and LDL heterogeneity

As noted in Garg's study, changes in LDL may not be as reliable as changes in other markers. Volek, *et al*. for example showed that whereas TAG was reduced in almost every subject on a low CHO diet, responses in LDL were highly variable [67]. The importance of LDL subpopulations has recently been appreciated and, unlike total LDL, changes in specific LDL particles show a consistent pattern with respect to dietary change.

**Figure 6:**
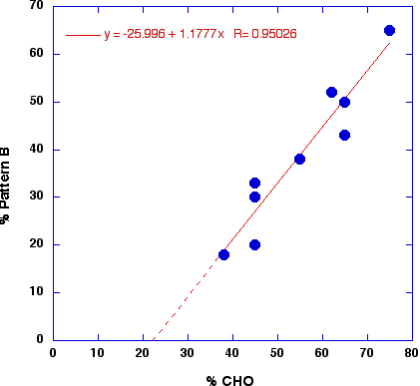
Prevalence of pattern B phenotype as a function of the percentage dietary CHO in men. Data from reference [71].

Greater atherogenic potential is associated with small, dense LDL particles [68]. Krauss and coworkers have carried out impressive work in defining the variability in different individuals. They identified a genetically influenced pattern (B) in people whose plasma contains small LDL particles. This subpopulation, typically 30 % of the American population, responded to low-fat diets by lowering LDL but the pattern B persisted [69]. The remaining subpopulation with larger buoyant particles (pattern A) responded to reduction in fat intake by a shift to the more atherogenic pattern B. Thus, for most of the populations studied, replacing dietary fat with CHO leads to a worsening of the LDL size distribution [70]. In a study described in reference [70], similar effects were seen when protein was substituted for carbohydrate without significant change in the fat content or composition. As summarized by Krauss, "This indicates that carbohydrate rather than fat is a major dietary determinant of expression or phenotype B in susceptible individuals." Although probably a semantic point, "susceptible" is redundant and describing pattern A and B as phenotypes may not be precise: Krauss has summarized how the relative amounts of CHO and fat affect the prevalence of pattern B [71] and the conclusion is that a strong relation exists between CHO intake (ranging from 40 to 75%) and the prevalence of the pattern B phenotype (Fig 6). In other words, there appears to be a continuous variability in phenotype characterized by sensitivity to CHO and everyone may be susceptible to conversion to pattern B at some CHO/fat ratio. The extrapolated line in Figure 6 suggests that a truly low CHO diet might reduce the level of atherogenic subtype to zero. Thus, whereas we have described the dialectic in practical applications as balancing the improvement in MetS with CHO restriction and the improvement in LDL from low-fat diets, focusing on LDL may have some caveats. In general, a growing body of work has shown improvement in LDL pattern switching from high CHO to low CHO diets [42,43,72-75].

The pattern B phenotype rarely occurs in isolation and, our major concern here is that it is metabolically linked to and co-expressed with other characteristics of MetS, particularly elevated TAG and low HDL. Krauss and colleagues reported that switching from a low CHO/high-fat diet (46% fat) to a high CHO/low-fat diet (26% fat) resulted in lowering of LDL, but also a worsening of TAG and HDL when switching to the low fat diet [69]. In men that were pattern A at a fat intake of 20 to 24%, a further reduction in dietary fat to 10% and CHO to 76% of energy resulted in conversion to B, with continued worsening of TAG and HDL, and no additional LDL-lowering [76].

### The TAG/HDL connection

In the search for markers for both insulin resistance and predisposition to CVD, recent research has focused on the value of the ratio of TAG:HDL. McLaughlin, et al. [8] have shown a correlation between insulin resistance as measured by steady-state glucose levels after infusion of glucose, insulin and octreotide (to suppress endogenous insulin secretion). The conclusions of their study were that TAG and HDL were independently related to insulin resistance and the TAG/HDL ratio was the best predictor of insulin resistance. Of importance here is that the results showed that this ratio is comparable to the ATP III criteria for MetS in predicting insulin resistance and "even better in prediction the LDL phenotype B in two separate populations who were on different diets." This is, in fact, only the most recent of several studies (references in [8]) that have shown a correlation between TAG/HDL and insulin resistance and CVD risk as measured by LDL particle size. Table 2-4 as well as Figures 2 and 5 indicate that low CHO diets reliably reduce this marker. Inability to recognize this is again due to the separate conditions in which they are measured and the continued emphasis on reducing dietary fat above all else.

McLaughlin's analysis [8] identified a TAG/HDL ratio of ≥ 3.5 as a cutoff for identifying the insulin-resistant patient most at risk for CVD. It is of interest that in Foster's study [45] described above (Table 3; Figure 1), the average beginning values were 4.6 and 4.3 in the low CHO and low-fat arms, respectively, substantially above this cutoff value. After six months, the low CHO arm had reduced this marker to 3.7 while the LF group showed little change at 4.2. Similarly, in the Delta-1 study, neither the Step 1 diet nor the low fat diet were able to improve the TAG/HDL ratio which was above the threshold value of 3.5 (Table 6)).

### Mechanism

A recent review by Ginsberg [77] has provided an excellent description of the possible mechanisms and central role of insulin resistance in mediating the dyslipidemia of MetS. In combination with a proposal by Volek [13] on the mechanism for the reversal of this process, a reasonable understanding of the connection between MetS and CHO restriction is possible.

A primary target of insulin is hormone-sensitive lipase. Adipocyte insulin resistance, plausibly a down regulation of insulin response due to continued stimulation (from higher dietary CHO), leads to increased lipolysis [78,79]. This will lead to greater delivery of fatty acids and an increase in hepatic esterification, and subsequent over production of VLDL, particularly the TAG-rich VLDL1. In combination with impaired plasma TAG clearance, a constant state of hypertriglyceridemia in the postabsorptive and postprandial period occurs. This leads to the exchange of TAG in VLDL for cholesteryl ester in LDL. The resulting TAG-rich LDL particle is a preferred substrate for hepatic lipase and lipoprotein lipase and thereby for generation of small, dense LDL. A similar neutral lipid exchange likely occurs with HDL whereby TAG-rich HDL is hydrolyzed by lipoprotein lipase resulting in the generation of smaller HDL particles that are rapidly removed from the circulation. In this way, elevated TAG resulting from disruption in insulin function, plays a central role in regulating the atherogenic dyslipidemia of MetS.

As noted above, Volek, et al. [13] have reviewed many studies showing that CHO restriction results in significant reductions in postprandial lipemia, and beneficial effects on HDL and intravascular processing of lipoproteins. A key component is what might be called the fatty acid paradox. Whereas insulin resistance is frequently characterized by high fatty acid levels, CHO restriction can improve insulin resistance while raising fatty acids. The latter effect is presumed to be due to lower insulin levels and disinhibition of hormone sensitive lipase. This is accompanied by enhanced cellular fatty acid uptake, mitochondrial transport and increased oxidation. The bias toward fat oxidation over storage reduces hepatic TAG and reduces synthesis and secretion of VLDL.

## Discussion

A joint position statement by several organizations recommended "current dietary guidelines from the ADA, AHA and the NCEP-ATP III.... These recommendations may require modification, however, as new information is generated from additional diet intervention studies [80]." Rather than additional studies, however, we provide new information from an evaluation of papers already in the literature that may provide a basis for modification. Data compiled in Figures 2, 3, 4 show that low CHO diets improve the symptoms of MetS as defined by five common criteria. We propose that in addition to potential value as therapy, the response to CHO restriction might be considered an operational definition of MetS. Beyond formal categorizing, the idea is consistent with the generally held belief that MetS is intimately involved with some form of insulin resistance. (The importance of other factors such as inflammation are not mutually exclusive). Cornier *et al*. showed differential benefit of low CHO vs. low fat diets for people with or without insulin resistance [51]; this study might be thought of as a model for future work to follow this line of thinking. We emphasize that our main point is that an intimate connection between CHO restriction and the complex of symptoms of MetS is seen in the literature of both low CHO studies and low fat (higher CHO) studies. Individual judgment as to the importance of MetS compared to other specific factors or more global assessments such as the Framingham criteria will determine how use is made of this connection. We do believe, however, that ignoring studies on CHO restriction would be unscientific and unproductive.

### How low is low carbohydrate?

*Goneril*. What need you five-and-twenty, ten, or five...?

*Regan*. What need one?

- William Shakespeare, **King Lear**.

The data summarized here suggest that some degree of CHO restriction would provide a first line of attack against the symptoms of MetS. The principle of CHO restriction is that by keeping insulin low, metabolism is biased towards lipid oxidation rather than storage, or the effects of fatty acids on peripheral tissues. Most studies that reported deleterious effects of saturated fat have been carried out in the presence of high CHO and there is a real question whether such effects carry over into hypocaloric conditions or those where insulin is better controlled [28,60,81,82].

In general, whereas current thinking in MetS emphasizes the consequences of insulin resistance, we feel that the role of CHO-induced hyperinsulinemia as a causative factor in generating the initial insulin resistance, dyslipidemia or obesity has been under-appreciated. In any case, there are now many ways to implement CHO restriction ranging from ketogenic diets (less than 50 g/d) to diets based on glycemic index, an indirect method of reducing insulin excursions. The question is how low is low? It is clear that 40% CHO is better than 55% for MetS and there has been some reluctance to go lower even though the studies that have done so show continued improvement. Perusal of Table 2-4 suggests that the lower, the better. Insofar as pattern B is associated with insulin resistance and MetS, examination of Figure 4 supports this idea. The barriers to exploring lower CHO diets appears to be continued emphasis on low fat intake although it has been known since Keys's Seven Country study that total fat in the diet does not correlate with cardiovascular risk [38]. At least as indicated in its popular diet book, the No-Fad Diet [83], the American Heart Association has removed its limitation on total fat which should open the door to more flexible diet interventions. Since many studies have shown that there is frequently a spontaneous reduction in total caloric intake in very low CHO diets and that CHO removed is not replaced by fat or protein, very low CHO now appears as a far more prudent choice than judged in the past.

### Is this new?

The phenomenon of CHO-induced hypertriglyceridemia is long established [84-88]. In addition, low fat diets are known to reduce, not only LDL, but also HDL levels. For example, the 2004 recommendations of the American Diabetic Association (ADA) state that "Low-saturated fat (i.e., 10% of energy) *high carbohydrate *diets *increase postprandial levels of plasma glucose, insulin, triglycerides *and, in some studies, *decrease plasma HDL cholesterol *when compared in metabolic studies to isocaloric high monounsaturated fat diets." This is our conclusion (our italics). We think the ADA statement could have been more clearly worded: "Substitution of CHO for monounsaturated fat "increase(s) postprandial levels of plasma glucose, insulin..." or could have been more comprehensive: "substitution of CHO or protein for fat increases..." In other words, it has been known for some time that low fat reduces HDL as well as LDL concentrations as described clearly in the Delta-1 study. Again, Rock's study that used the increase in TAG and decrease in HDL as a marker for compliance to a low fat diet supports the idea as an accepted principle.

In combination with the experimentally observed and intuitively obvious reduction in fasting blood glucose and insulin, our proposal for the importance of CHO replacement in the diet hardly seems new. Yet such an idea, to our knowledge has never been made explicit. The primacy of the low fat paradigm in traditional thinking may have played a role in ignoring this obvious correlation. However, we have, in various places, presented or summarized evidence that low CHO diets have a beneficial effect on MetS [13,16] but the tight connection proposed here conceptually eluded us. Having raised the question, however, it is now clear that it is perfectly consistent with established knowledge.

### Is MetS useful?

The intricacies of the debate among official agencies on the clinical importance of MetS [9] are beyond the scope of this article. As a first order approximation, however, we are inclined to follow Reaven's strategy in the Point/Counterpoint paper [10,89] for assessing the need for the concept of MetS. He describes a patient with a BMI of 27.8 kg/m^2 ^who would not conform to the WHO definition of MetS because of acceptable values of glucose, TAG and an HDL level of 37 mg/dL and he points out that if the HDL level fell to 33 mg/dL such a patent would fit a new criteria but would not sensibly be treated in a different way. Under the approach considered here, the patient with the higher HDL would be presented with a number of options for weight loss whereas the patient with lower HDL might reasonably be counseled to CHO restriction as a first strategy. We therefore think that MetS or some combination of markers by any other name would have much more virility than the description of MetS as Methuselah in his amusing Counterpoint [87]. We also side with the "Pro" position on the value for basic research. Reaven raises the critical question: "How can there be a common etiology for a diagnostic category based on satisfying 3 of 5 arbitrarily defined criteria when any combination of the 3 will define the same phenotype as any other trio of abnormalities.?" A plausible answer to this question is that if all the markers in MetS are related to hyperinsulinemia and/or insulin resistance, then the relative K_m_'s for insulin for the different target proteins and different tissues are likely to lead to a variable time course for specific individuals and markers are likely to exceed cutoff at different times for each patient. Appearance of one marker may then be indicative of other still silent conditions.

### Recommendations for the American population

The data summarized here suggest that there is value in the definition of MetS and that a nutritional strategy based on CHO restriction might sensibly be the "default" diet, the first to be tried, for patients with MetS. In the case of normal weight individuals with MetS, CHO restriction may be the only effective non-pharmacological approach for treating the diversity of symptoms. The choice of any intervention, however, depends on individual assessment of the relative importance of different risk factors and our goal here is the establishment of the close link between CHO restriction and MetS rather than any recommendation.

The recent AHA/NHLBI Scientific Statement on Metabolic Syndrome [90,91] as well as the ATP III emphasize as the primary target, LDL, a marker that is not considered a feature of MetS and that may not even be high in many patients with MetS. Whereas nutritional recommendations are quite general (reduce weight, increase exercise), these reports emphasize a low fat diet (although limiting simple sugars). We think this is inconsistent and we have made the case that a low fat diet, if high in carbohydrate, seems to be widely accepted as raising TAG, lowering HDL and worsening glycemic control, seemingly the wrong thing for MetS. We also disagree with the assertion in the AHA/NHLBI statement that most low CHO diets are high in saturated fat. This statement is undocumented and, as noted in the introduction, essentially equates all reduced carbohydrate approaches and does not seem to distinguish between total and saturated fat. Even if it were true the statement avoids the question of the effect of saturated fat in the presence of low CHO or hypocaloric diets [60,81,82,92]. In practical terms, a recommendation to reduce saturated fat on low CHO diets might be more helpful than blanket prohibition. In addition, the AHA/NHLBI report [90] presents the rationale for low CHO as the effect on appetite. Whereas this may be a component, it has been stated many times, and is part of basic biochemical education [16,93-96], that the rationale of CHO restriction is the control of metabolism by insulin regulation. The effects described above clearly support this. Although we think that, within the framework of MetS, some recommendations can be made, in the end we are probably in agreement with Reaven's judgment that "What is required is less advice and more information [54]."

### Questions raised

The data summarized here leave little room for doubt that the generally accepted deterioration in HDL and TAG levels with low fat diets and the established improvement in glycemic control with CHO restriction are part of a more general picture. The hypothesis that response to CHO restriction (because of the effect on insulin) is the defining feature of MetS is the proposed generalization. This idea raises several questions.

Is there a threshold level of CHO restriction that is necessary to elicit improvements in MetS? What is the effect of replacing the calories lost from CHO with protein or with fat, and in what proportion? How does this compare to not replacing them at all?

Does excessive CHO consumption *cause *MetS in susceptible individuals?

What is the relative risk in addressing MetS with CHO restriction compared to low fat diets for reduction in LDL? That is, what is the relative risk of high LDL vs. the symptoms that comprise MetS?

What is the role of genotype in determining the response to CHO restriction?

## Summary

Five symptoms common to most definitions of MetS are those that are reliably improved by CHO restriction. Carbohydrate restriction is one strategy for weight loss but, in addition, improves glycemic control, insulin levels, TAG and HDL levels even in the absence of weight loss. We suggest that response to CHO restriction may, in fact, be an operational definition of MetS. Its underlying basis would rest on the idea that the features of MetS are associated with a disruption in insulin metabolism which is strongly influenced by dietary CHO. The extent to which this definition is useful may depend on its application by individual practitioners. Experimental studies that follow its lead or conversely disprove its fundamental premise should advance our understanding of obesity, diabetes and CVD. Dismissing CHO restriction without evidence, or expressing "concerns" rather than offering data will probably be less productive.

## Abbreviations

Adult Treatment Panel (ATP III), American Diabetes Association (ADA), American Heart Association (AHA), Average American diet (AAD), carbohydrate (CHO), low fat (LF), Metabolic Syndrome (MetS), National Cholesterol Education Program (NCEP), triacylglycerol (TAG), World Health Organization (WHO)

## Competing interests

The author(s) declare that they have no competing interests.

## Note

**Table 2 - Effect of carbohydrate restriction on markers for Metabolic Syndrome **(See Table 2)

Data shown in bold indicate improvement in marker, plain, worsening. References:1. [97]; 2. [24]; 3. [98]; 4. [99]; 5. [100]; 6. [101]; 7. [42]; 8. [102]; 9. [103]; 10. [104]; 11. [74]; 12. [105]; 13. [23].

**Table 3 - Effect of carbohydrate restriction on markers for Metabolic Syndrome under conditions of constant body mass **(See Table 3)

Data shown in bold indicate low CHO shows greater improvement in markers for MetS than LF; plain, LF is better. Table reference 3 shows the ratio of low CHO to LF. References: 1. [42]; 2. [43]; 3. [44].

**Table 4 - Comparison of low CHO vs. LF diets on markers for Metabolic Syndrome **(See Table 4)

Data shown in bold indicate low CHO (or mod-PROT) shows greater improvement in marker than LF; plain, LF is better. Experiment in reference [106] was carried out for a longer time period but diets became very similar. References:1. [107]; 2. [113]; 3. [46]; 4. [45]; 5. [108]; 6. [72]; 7. [109]; 8. [110]; 9. [111]; 10. [112]; 11. [73]; 12. [75]; 13. [59]; 14. [106].

**Table 6 - Outcomes of the Delta-1 study **(See Table 6)

Data from reference [64]. bold indicates improvement in the parameter from Step 1 or low Saturated Fat diet compared to AAD; plain indicates worsening of parameter compared to AAD.

## References

[B1] Eckel RH Grundy SM Zimmet PZ The metabolic syndrome Lancet 2005 365 9468 1415 1428 10.1016/S0140-6736(05)66378-7 15836891

[B2] Grundy SM Brewer HBJ Cleeman JI Smith SCJ Lenfant C Definition of metabolic syndrome: Report of the National Heart, Lung, and Blood Institute/American Heart Association conference on scientific issues related to definition Circulation 2004 109 3 433 438 10.1161/01.CIR.0000111245.75752.C6 14744958

[B3] Grundy SM Hansen B Smith SCJ Cleeman JI Kahn RA Clinical management of metabolic syndrome: report of the American Heart Association/National Heart, Lung, and Blood Institute/American Diabetes Association conference on scientific issues related to management Arterioscler Thromb Vasc Biol 2004 24 2 e19 24 10.1161/01.ATV.0000112379.88385.67 14766740

[B4] Tonkin A The metabolic syndrome -- a growing problem European Heart Journal Supplements 2004 6 A37 A42

[B5] Aude YW Mego P Mehta JL Metabolic syndrome: dietary interventions Curr Opin Cardiol 2004 19 5 473 479 10.1097/01.hco.0000134610.68815.05 15316456

[B6] Hanley AJ Wagenknecht LE D'Agostino RBJ Zinman B Haffner SM Identification of subjects with insulin resistance and beta-cell dysfunction using alternative definitions of the metabolic syndrome Diabetes 2003 52 11 2740 2747 14578292 10.2337/diabetes.52.11.2740

[B7] Hunt KJ Resendez RG Williams K Haffner SM Stern MP National Cholesterol Education Program versus World Health Organization metabolic syndrome in relation to all-cause and cardiovascular mortality in the San Antonio Heart Study Circulation 2004 110 10 1251 1257 10.1161/01.CIR.0000140762.04598.F9 15326061

[B8] McLaughlin T Reaven G Abbasi F Lamendola C Saad M Waters D Simon J Krauss RM Is there a simple way to identify insulin-resistant individuals at increased risk of cardiovascular disease? Am J Cardiol 2005 96 3 399 404 10.1016/j.amjcard.2005.03.085 16054467

[B9] Kahn R Buse J Ferrannini E Stern M The metabolic syndrome: time for a critical appraisal: joint statement from the American Diabetes Association and the European Association for the Study of Diabetes Diabetes Care 2005 28 9 2289 2304 16123508 10.2337/diacare.28.9.2289

[B10] Grundy SM Point: the metabolic syndrome still lives Clin Chem 2005 51 8 1352 1354 10.1373/clinchem.2005.050989 16040840

[B11] Reaven GM The metabolic syndrome: requiescat in pace Clin Chem 2005 51 6 931 938 10.1373/clinchem.2005.048611 15746300

[B12] Arora SK McFarlane SI The case for low carbohydrate diets in diabetes management Nutr Metab (Lond) 2005 2 16 10.1186/1743-7075-2-16 16018812 PMC1188071

[B13] Volek JS Sharman MJ Forsythe CE Modification of lipoproteins by very low-carbohydrate diets J Nutr 2005 135 6 1339 1342 15930434 10.1093/jn/135.6.1339

[B14] Third Report of the National Cholesterol Education Program (NCEP) Expert Panel on Detection, Evaluation, and Treatment of High Blood Cholesterol in Adults (Adult Treatment Panel III) final report Circulation 2002 106 25 3143 3421 12485966

[B15] Ashen MD Blumenthal RS Clinical practice. Low HDL cholesterol levels N Engl J Med 2005 353 12 1252 1260 10.1056/NEJMcp044370 16177251

[B16] Feinman RD Makowske M Metabolic Syndrome and Low-Carbohydrate Ketogenic Diets in the Medical School Biochemistry Curriculum. Metabolic Syndrome and Related Disorders 2003 1 189 198 10.1089/154041903322716660 18370662

[B17] Westman EC Yancy Jr. WS Haub MD Volek JS Insulin Resistance from a Low-Carbohydrate, High Fat Diet Perspective Metabolic Syndrome and Related Disorders 2005 3 3 7 10.1089/met.2005.3.14 18370705

[B18] Ruderman N Chisholm D Pi-Sunyer X Schneider S The metabolically obese, normal-weight individual revisited Diabetes 1998 47 5 699 713 9588440 10.2337/diabetes.47.5.699

[B19] Willet W Increasing prevalence of overweight among US adults. The National Health and Nutrition Examination Surveys, 1960 to 1991 JAMA 1994 272 205 211 10.1001/jama.272.3.205 8022039

[B20] Poppitt SD Keogh GF Prentice AM Williams DE Sonnemans HM Valk EE Robinson E Wareham NJ Long-term effects of ad libitum low-fat, high-carbohydrate diets on body weight and serum lipids in overweight subjects with metabolic syndrome Am J Clin Nutr 2002 75 1 11 20 11756055 10.1093/ajcn/75.1.11

[B21] Enns CK Goldman JD Cook A Trends in Food and Nutrient Intakes by Adults: NFCS 1977-78, CSFII 1989-91, and CSFII 1994-95 Family Economics and Nutrition Review 1997 10 2 15

[B22] Kennedy ET Bowman SA Powell R Dietary-fat intake in the US population J Am Coll Nutr 1999 18 3 207 212 10376775 10.1080/07315724.1999.10718853

[B23] Boden G Sargrad K Homko C Mozzoli M Stein TP Effect of a low-carbohydrate diet on appetite, blood glucose levels, and insulin resistance in obese patients with type 2 diabetes Ann Intern Med 2005 142 6 403 411 15767618 10.7326/0003-4819-142-6-200503150-00006

[B24] Larosa JC Fry AG Muesing R Rosing DR Effects of high-protein, low-carbohydrate dieting on plasma lipoproteins and body weight J Am Diet Assoc 1980 77 3 264 270 7410754

[B25] Miller BV Bertino J Reed TG Burrington C Davidson LK Green A Gartung A Nafziger A An Evaluation of the Atkins' Diet Metabolic Syndrome and Related Disorders 2003 1 299 309 10.1089/1540419031361426 18370655

[B26] Bray GA The epidemic of obesity and changes in food intake: the Fluoride Hypothesis Physiol Behav 2004 82 1 115 121 10.1016/j.physbeh.2004.04.033 15234599

[B27] Martin WF Armstrong LE Rodriguez NR Dietary Protein Intake and Renal Function Nutr Metab (Lond) 2005 2 1 25 10.1186/1743-7075-2-25 16174292 PMC1262767

[B28] Van der Auwera I Wera S Van Leuven F Henderson ST A ketogenic diet reduces amyloid beta 40 and 42 in a mouse model of Alzheimer's disease Nutr Metab (Lond) 2005 10.1186/1743-7075-2-28 PMC1282589 16229744

[B29] Veech RL The therapeutic implications of ketone bodies: the effects of ketone bodies in pathological conditions: ketosis, ketogenic diet, redox states, insulin resistance, and mitochondrial metabolism Prostaglandins Leukot Essent Fatty Acids 2004 70 3 309 319 10.1016/j.plefa.2003.09.007 14769489

[B30] Veech RL Chance B Kashiwaya Y Lardy HA Cahill GFJ Ketone bodies, potential therapeutic uses IUBMB Life 2001 51 4 241 247 11569918 10.1080/152165401753311780

[B31] Brand-Miller JC Glycemic index in relation to coronary disease Asia Pac J Clin Nutr 2004 13 Suppl S315294465

[B32] Brand-Miller JC Postprandial glycemia, glycemic index, and the prevention of type 2 diabetes Am J Clin Nutr 2004 80 2 243 244 15277141 10.1093/ajcn/80.2.243

[B33] Atkins RC Dr. Atkins' New Diet Revolution 2002 New York , Avon Books

[B34] Agatston A The South Beach Diet 2003 New York , Random House

[B35] Eades MR Eades MD Protein Power 1996 New York , Bantam Books

[B36] Hu FB Manson JE Willett WC Types of dietary fat and risk of coronary heart disease: a critical review J Am Coll Nutr 2001 20 1 5 19 11293467 10.1080/07315724.2001.10719008

[B37] Hu FB Stampfer MJ Manson JE Rimm E Colditz GA Rosner BA Hennekens CH Willett WC Dietary fat intake and the risk of coronary heart disease in women N Engl J Med 1997 337 21 1491 1499 10.1056/NEJM199711203372102 9366580

[B38] Keys A Coronary heart disease in seven countries 1970 41 (Suppl) 1 211 10.1016/s0899-9007(96)00410-8 9131696

[B39] Feinman RD Fine EJ Thermodynamics and Metabolic Advantage of Weight Loss Diets. Metabolic Syndrome and Related Disorders 2003 1 209 219 10.1089/154041903322716688 18370664

[B40] Volek JS Westman EC Very-low-carbohydrate weight-loss diets revisited Cleve Clin J Med 2002 69 11 849, 853, 856 8 passim 12430970 10.3949/ccjm.69.11.849

[B41] Westman EC Mavropoulos J Yancy WS Volek JS A Review of Low-carbohydrate Ketogenic Diets Curr Atheroscler Rep 2003 5 6 476 483 14525681 10.1007/s11883-003-0038-6

[B42] Sharman MJ Kraemer WJ Love DM Avery NG Gomez AL Scheett TP Volek JS A ketogenic diet favorably affects serum biomarkers for cardiovascular disease in normal-weight men J Nutr 2002 132 7 1879 1885 12097663 10.1093/jn/132.7.1879

[B43] Volek JS Sharman MJ Gomez AL Scheett TP Kraemer WJ An Isoenergetic Very Low-Carbohydrate Diet Is Associated With Improved Serum High-Density Lipoprotein Cholesterol (HDL-C), Total Cholesterol to HDL-C Ratio, Triacylglycerols, and Postprandial Lipemic Responses Compared to a Low-Fat Diet in Normal Weight, Normolipidemic Women J Nutr 2003 133 9 2756 2761 12949361 10.1093/jn/133.9.2756

[B44] Allick G Bisschop PH Ackermans MT Endert E Meijer AJ Kuipers F Sauerwein HP Romijn JA A low-carbohydrate/high-fat diet improves glucoregulation in type 2 diabetes mellitus by reducing postabsorptive glycogenolysis J Clin Endocrinol Metab 2004 89 12 6193 6197 10.1210/jc.2004-1041 15579777

[B45] Foster GD Wyatt HR Hill JO McGuckin BG Brill C Mohammed BS Szapary PO Rader DJ Edman JS Klein S A randomized trial of a low-carbohydrate diet for obesity N Engl J Med 2003 348 21 2082 2090 10.1056/NEJMoa022207 12761365

[B46] Samaha FF Iqbal N Seshadri P Chicano KL Daily DA McGrory J Williams T Williams M Gracely EJ Stern L A low-carbohydrate as compared with a low-fat diet in severe obesity N Engl J Med 2003 348 21 2074 2081 10.1056/NEJMoa022637 12761364

[B47] Feinman RD Fine EJ "A Calorie is a calorie" violates the second law of thermodynamics. Nutrition Journal 2004 3 9 10.1186/1475-2891-3-9 PMC506782 15282028

[B48] Feinman RD Fine EJ Whatever happened to the second law of thermodynamics? Am J Clin Nutr 2004 80 5 1445 6; author reply 1446 15531699 10.1093/ajcn/80.5.1445

[B49] Fine EJ Feinman RD Thermodynamics of weight loss diets Nutr Metab (Lond) 2004 1 1 15 10.1186/1743-7075-1-15 15588283 PMC543577

[B50] Bray GA Champagne CM Beyond energy balance: there is more to obesity than kilocalories J Am Diet Assoc 2005 105 5 Suppl 1 S17 23 10.1016/j.jada.2005.02.018 15867891

[B51] Cornier MA Donahoo WT Pereira R Gurevich I Westergren R Enerback S Eckel PJ Goalstone ML Hill JO Eckel RH Draznin B Insulin sensitivity determines the effectiveness of dietary macronutrient composition on weight loss in obese women Obes Res 2005 13 4 703 709 15897479 10.1038/oby.2005.79

[B52] Golay A Eigenheer C Morel Y Kujawski P Lehmann T de Tonnac N Weight-loss with low or high carbohydrate diet? Int J Obes Relat Metab Disord 1996 20 12 1067 1072 8968851

[B53] Poe EA The Purloined Letter http://xroadsvirginiaedu/~HYPER/POE/purloinehtml 1845

[B54] Reaven GM Effect of dietary carbohydrate on the metabolism of patients with non-insulin dependent diabetes mellitus Nutr Rev 1986 44 2 65 73 3703391 10.1111/j.1753-4887.1986.tb07589.x

[B55] Gannon MC Nuttall FQ Saeed A Jordan K Hoover H An increase in dietary protein improves the blood glucose response in persons with type 2 diabetes Am J Clin Nutr 2003 78 4 734 741 14522731 10.1093/ajcn/78.4.734

[B56] Nuttall FQ Gannon MC Metabolic response of people with type 2 diabetes to a high protein diet Nutr Metab (Lond) 2004 1 1 6 10.1186/1743-7075-1-6 15507157 PMC524031

[B57] Gannon MC Nuttall FQ Effect of a high-protein, low-carbohydrate diet on blood glucose control in people with type 2 diabetes Diabetes 2004 53 9 2375 2382 15331548 10.2337/diabetes.53.9.2375

[B58] Ludwig DS Jenkins DJ Carbohydrates and the postprandial state: have our cake and eat it too? Am J Clin Nutr 2004 80 4 797 798 15447882 10.1093/ajcn/80.4.797

[B59] McAuley KA Hopkins CM Smith KJ McLay RT Williams SM Taylor RW Mann JI Comparison of high-fat and high-protein diets with a high-carbohydrate diet in insulin-resistant obese women Diabetologia 2005 48 1 8 16 10.1007/s00125-004-1603-4 15616799

[B60] Feinman R When is a high fat diet not a high fat diet? Nutrition & Metabolism 2005 2 27 10.1186/1743-7075-2-27 PMC1283748 16229741

[B61] Garg A Bantle JP Henry RR Coulston AM Griver KA Raatz SK Brinkley L Chen YD Grundy SM Huet BA Effects of varying carbohydrate content of diet in patients with non-insulin-dependent diabetes mellitus Jama 1994 271 18 1421 1428 10.1001/jama.271.18.1421 7848401

[B62] Mensink RP Zock PL Kester AD Katan MB Effects of dietary fatty acids and carbohydrates on the ratio of serum total to HDL cholesterol and on serum lipids and apolipoproteins: a meta-analysis of 60 controlled trials Am J Clin Nutr 2003 77 5 1146 1155 12716665 10.1093/ajcn/77.5.1146

[B63] Rock CL Flatt SW Thomson CA Stefanick ML Newman VA Jones L Natarajan L Pierce JP Chang RJ Witztum JL Plasma triacylglycerol and HDL cholesterol concentrations confirm self-reported changes in carbohydrate and fat intakes in women in a diet intervention trial J Nutr 2004 134 2 342 347 14747670 10.1093/jn/134.2.342

[B64] Ginsberg HN Kris-Etherton P Dennis B Elmer PJ Ershow A Lefevre M Pearson T Roheim P Ramakrishnan R Reed R Stewart K Stewart P Phillips K Anderson N Effects of reducing dietary saturated fatty acids on plasma lipids and lipoproteins in healthy subjects: the DELTA Study, protocol 1 Arterioscler Thromb Vasc Biol 1998 18 3 441 449 9514413 10.1161/01.atv.18.3.441

[B65] Gordon DJ Knoke J Probstfield JL Superko R Tyroler HA High-density lipoprotein cholesterol and coronary heart disease in hypercholesterolemic men: the Lipid Research Clinics Coronary Primary Prevention Trial Circulation 1986 74 1217 1225 3536151 10.1161/01.cir.74.6.1217

[B66] Berglund L Oliver EH Fontanez N Holleran S Matthews K Roheim PS Ginsberg HN Ramakrishnan R Lefevre M HDL-subpopulation patterns in response to reductions in dietary total and saturated fat intakes in healthy subjects Am J Clin Nutr 1999 70 6 992 1000 10584043 10.1093/ajcn/70.6.992

[B67] Volek JS Sharman MJ Cardiovascular and hormonal aspects of very-low-carbohydrate ketogenic diets Obes Res 2004 12 Suppl 2 115S 23S 15601959 10.1038/oby.2004.276

[B68] Lamarche B Tchernof A Moorjani S Cantin B Dagenais GR Lupien PJ Despres JP Small, dense low-density lipoprotein particles as a predictor of the risk of ischemic heart disease in men. Prospective results from the Quebec Cardiovascular Study Circulation 1997 95 1 69 75 8994419 10.1161/01.cir.95.1.69

[B69] Dreon DM Fernstrom HA Miller B Krauss RM Low-density lipoprotein subclass patterns and lipoprotein response to a reduced-fat diet in men Faseb J 1994 8 1 121 126 8299884

[B70] Krauss RM Dietary and Genetic Probes of Atherogenic Dyslipidemia Arterioscler Thromb Vasc Biol 2005 10.1161/01.ATV.0000186365.73973.f0 16166563

[B71] Krauss RM Atherogenic lipoprotein phenotype and diet-gene interactions J Nutr 2001 131 2 340S 3S 11160558 10.1093/jn/131.2.340S

[B72] Sharman MJ Gomez AL Kraemer WJ Volek JS Very low-carbohydrate and low-fat diets affect fasting lipids and postprandial lipemia differently in overweight men J Nutr 2004 134 4 880 885 15051841 10.1093/jn/134.4.880

[B73] Aude YW Agatston AS Lopez-Jimenez F Lieberman EH Marie A Hansen M Rojas G Lamas GA Hennekens CH The national cholesterol education program diet vs a diet lower in carbohydrates and higher in protein and monounsaturated fat: a randomized trial Arch Intern Med 2004 164 19 2141 2146 10.1001/archinte.164.19.2141 15505128

[B74] Hays JH DiSabatino A Gorman RT Vincent S Stillabower ME Effect of a high saturated fat and no-starch diet on serum lipid subfractions in patients with documented atherosclerotic cardiovascular disease Mayo Clin Proc 2003 78 11 1331 1336 14601690 10.4065/78.11.1331

[B75] Seshadri P Iqbal N Stern L Williams M Chicano KL Daily DA McGrory J Gracely EJ Rader DJ Samaha FF A randomized study comparing the effects of a low-carbohydrate diet and a conventional diet on lipoprotein subfractions and C-reactive protein levels in patients with severe obesity Am J Med 2004 117 6 398 405 10.1016/j.amjmed.2004.04.009 15380496

[B76] Dreon DM Fernstrom HA Williams PT Krauss RM A very low-fat diet is not associated with improved lipoprotein profiles in men with a predominance of large, low-density lipoproteins Am J Clin Nutr 1999 69 3 411 418 10075324 10.1093/ajcn/69.3.411

[B77] Ginsberg HN Zhang YL Hernandez-Ono A Regulation of plasma triglycerides in insulin resistance and diabetes Arch Med Res 2005 36 3 232 240 10.1016/j.arcmed.2005.01.005 15925013

[B78] Boden G Shulman GI Free fatty acids in obesity and type 2 diabetes: defining their role in the development of insulin resistance and beta-cell dysfunction Eur J Clin Invest 2002 32 Suppl 3 14 23 10.1046/j.1365-2362.32.s3.3.x 12028371

[B79] Zammit VA Insulin stimulation of hepatic triacylglycerol secretion in the insulin-replete state: implications for the etiology of peripheral insulin resistance Ann N Y Acad Sci 2002 967 52 65 12079835 10.1111/j.1749-6632.2002.tb04263.x

[B80] Klein S Burke LE Bray GA Blair S Allison DB Pi-Sunyer X Hong Y Eckel RH Clinical implications of obesity with specific focus on cardiovascular disease: a statement for professionals from the American Heart Association Council on Nutrition, Physical Activity, and Metabolism: endorsed by the American College of Cardiology Foundation Circulation 2004 110 18 2952 2967 10.1161/01.CIR.0000145546.97738.1E 15509809

[B81] Mozaffarian D Rimm EB Herrington DM Dietary fats, carbohydrate, and progression of coronary atherosclerosis in postmenopausal women Am J Clin Nutr 2004 80 5 1175 1184 15531663 10.1093/ajcn/80.5.1175 PMC1270002

[B82] Volek JS Forsythe CE The case for not restricting saturated fat on a low carbohydrate diet Nutr Metab (Lond) 2005 2 21 10.1186/1743-7075-2-21 16135250 PMC1208952

[B83] American Heart Association No-Fad Diet. A Personal Plan fo Healthy Weight Loss 2005 New York , Clarkson Potter

[B84] Hellerstein MK Carbohydrate-induced hypertriglyceridemia: modifying factors and implications for cardiovascular risk Curr Opin Lipidol 2002 13 1 33 40 10.1097/00041433-200202000-00006 11790961

[B85] Hudgins LC Effect of high-carbohydrate feeding on triglyceride and saturated fatty acid synthesis Proc Soc Exp Biol Med 2000 225 3 178 183 10.1046/j.1525-1373.2000.22521.x 11082210

[B86] Hudgins LC Hellerstein M Seidman C Neese R Diakun J Hirsch J Human fatty acid synthesis is stimulated by a eucaloric low fat, high carbohydrate diet J Clin Invest 1996 97 9 2081 2091 8621798 10.1172/JCI118645 PMC507283

[B87] Parks EJ Hellerstein MK Carbohydrate-induced hypertriacylglycerolemia: historical perspective and review of biological mechanisms Am J Clin Nutr 2000 71 2 412 433 10648253 10.1093/ajcn/71.2.412

[B88] Parks EJ Krauss RM Christiansen MP Neese RA Hellerstein MK Effects of a low-fat, high-carbohydrate diet on VLDL-triglyceride assembly, production, and clearance J Clin Invest 1999 104 8 1087 1096 10525047 10.1172/JCI6572 PMC408572

[B89] Reaven G Counterpoint: just being alive is not good enough Clin Chem 2005 51 8 1354 1357 10.1373/clinchem.2005.053587 16040841

[B90] Grundy SM Cleeman JI Daniels SR Donato KA Eckel RH Franklin BA Gordon DJ Krauss RM Savage PJ Smith SCJ Spertus JA Costa F Diagnosis and Management of the Metabolic Syndrome. An American Heart Association/National Heart, Lung, and Blood Institute Scientific Statement Circulation 2005 10.1161/CIRCULATIONAHA.105.169404 16157765

[B91] Grundy SM Cleeman JI Daniels SR Donato KA Eckel RH Franklin BA Gordon DJ Krauss RM Savage PJ Smith SCJ Spertus JA Costa F Diagnosis and Management of the Metabolic Syndrome. An American Heart Association/National Heart, Lung, and Blood Institute Scientific Statement. Executive Summary Circulation 2005 10.1097/00132577-200512000-00018 18340209

[B92] Knopp RH Retzlaff BM Saturated fat prevents coronary artery disease? An American paradox Am J Clin Nutr 2004 80 5 1102 1103 15531654 10.1093/ajcn/80.5.1102

[B93] Pogozelski W Arpaia N Priore S The Metabolic Effects of Low-carbohydrate Diets and Incorporation into a Biochemistry Course Biochemistry and Molecular Biology Education 2005 33 91 100 21638552 10.1002/bmb.2005.494033022445

[B94] Harris RA Crabb DW Devlin TM Chapter 22. Metabolic Interrelationships Textbook of Biochemistry With Clinical Correlations 2006 Sixth New York , John Wiley & Sons, Inc.

[B95] Makowske M Feinman RD Nutrition education: a questionnaire for assessment and teaching Nutr J 2005 4 1 2 10.1186/1475-2891-4-2 15649324 PMC546238

[B96] Smith C Marks AD Lieberman M Basic Medical Biochemistry: A Clinical Approach 2005 2nd Philadelphia , Lippincott Williams & Wilkins

[B97] Rickman F Mitchell N Dingman J Dalen JE Changes in serum cholesterol during the Stillman diet Jama 1974 228 1 54 58 10.1001/jama.228.1.54 4406145

[B98] Phinney SD Horton ES Sims EA Hanson JS Danforth EJ LaGrange BM Capacity for moderate exercise in obese subjects after adaptation to a hypocaloric, ketogenic diet J Clin Invest 1980 66 5 1152 1161 7000826 10.1172/JCI109945 PMC371554

[B99] Phinney SD Bistrian BR Evans WJ Gervino E Blackburn GL The human metabolic response to chronic ketosis without caloric restriction: preservation of submaximal exercise capability with reduced carbohydrate oxidation Metabolism 1983 32 8 769 776 10.1016/0026-0495(83)90106-3 6865776

[B100] Newbold HL Reducing the serum cholesterol level with a diet high in animal fat South Med J 1988 81 1 61 63 3336803 10.1097/00007611-198801000-00013

[B101] Volek JS Gomez AL Kraemer WJ Fasting lipoprotein and postprandial triacylglycerol responses to a low-carbohydrate diet supplemented with n-3 fatty acids J Am Coll Nutr 2000 19 3 383 391 10872901 10.1080/07315724.2000.10718935

[B102] Meckling KA Gauthier M Grubb R Sanford J Effects of a hypocaloric, low-carbohydrate diet on weight loss, blood lipids, blood pressure, glucose tolerance, and body composition in free-living overweight women Can J Physiol Pharmacol 2002 80 11 1095 1105 10.1139/y02-140 12489929

[B103] Westman EC Yancy WS Edman JS Tomlin KF Perkins CE Effect of 6-month adherence to a very low carbohydrate diet program Am J Med 2002 113 1 30 36 10.1016/S0002-9343(02)01129-4 12106620

[B104] Dashti HM Bo-Abbas YY Asfar SK Mathew TC Hussein T Behbahani A Khoursheed MA Al-Sayer HM Al-Zaid NS Ketogenic diet modifies the risk factors of heart disease in obese patients Nutrition 2003 19 10 901 902 10.1016/S0899-9007(03)00161-8 14559328

[B105] Dashti HM Mathew TC Hussein T Asfar SK Behbahani A Khoursheed MA Al-Sayer HM Bo-Abbas YY Al-Zaid NS Long-term effects of a ketogenic diet in obese patients Exp Clin Cardiol 2004 9 3 200 205 19641727 PMC2716748

[B106] Dansinger ML Gleason JA Griffith JL Selker HP Schaefer EJ Comparison of the Atkins, Ornish, Weight Watchers, and Zone diets for weight loss and heart disease risk reduction: a randomized trial Jama 2005 293 1 43 53 10.1001/jama.293.1.43 15632335

[B107] Brehm BJ Seeley RJ Daniels SR D'Alessio DA A randomized trial comparing a very low carbohydrate diet and a calorie-restricted low fat diet on body weight and cardiovascular risk factors in healthy women J Clin Endocrinol Metab 2003 88 4 1617 1623 10.1210/jc.2002-021480 12679447

[B108] Volek JS Sharman MJ Gomez AL Judelson DA Rubin MR Watson G Sokmen B Silvestre R French DN Kraemer WJ Comparison of energy-restricted very low-carbohydrate and low-fat diets on weight loss and body composition in overweight men and women Nutr Metab (Lond) 2004 1 1 13 10.1186/1743-7075-1-13 15533250 PMC538279

[B109] Brehm BJ Spang SE Lattin BL Seeley RJ Daniels SR D'Alessio DA The role of energy expenditure in the differential weight loss in obese women on low-fat and low-carbohydrate diets J Clin Endocrinol Metab 2004 10.1210/jc.2004-1540 15598683

[B110] Meckling KA O'Sullivan C Saari D Comparison of a low-fat diet to a low-carbohydrate diet on weight loss, body composition, and risk factors for diabetes and cardiovascular disease in free-living, overweight men and women J Clin Endocrinol Metab 2004 89 6 2717 2723 10.1210/jc.2003-031606 15181047

[B111] Stern L Iqbal N Seshadri P Chicano KL Daily DA McGrory J Williams M Gracely EJ Samaha FF The effects of low-carbohydrate versus conventional weight loss diets in severely obese adults: one-year follow-up of a randomized trial Ann Intern Med 2004 140 10 778 785 15148064 10.7326/0003-4819-140-10-200405180-00007

[B112] Yancy WSJ Olsen MK Guyton JR Bakst RP Westman EC A low-carbohydrate, ketogenic diet versus a low-fat diet to treat obesity and hyperlipidemia: a randomized, controlled trial Ann Intern Med 2004 140 10 769 777 15148063 10.7326/0003-4819-140-10-200405180-00006

